# Carbonation and Chloride Resistance Characteristics of Self-Developed Limestone Calcined Clay Cement (LC3) Derived from Excavated Spoil

**DOI:** 10.3390/ma18112546

**Published:** 2025-05-28

**Authors:** Yunyuan Li, Lixin Miao, Zhijun Dong, Yu Jin, Wei Liu, Fangsheng Gao, Yongqiang Li

**Affiliations:** 1 Tsinghua Shenzhen International Graduate School, Tsinghua Campus, The University Town, Shenzhen 518055, China; li-yy22@mails.tsinghua.edu.cn (Y.L.); lxmiao@tsinghua.edu.cn (L.M.); 2 Institute of Technology for Future Industry, Shenzhen Institute of Information Technology, Shenzhen 518172, China; dongzj@sziit.edu.cn (Z.D.); 2018000046@sziit.edu.cn (Y.J.); 3 Guangdong Provincial Key Laboratory of Durability for Marine Civil Engineering, College of Civil and Transportation Engineering, Shenzhen University, Shenzhen 518060, China; liuwei@szu.edu.cn; 4 Shenzhen Antuoshan Concrete Co., Ltd., Shenzhen 518060, China; gfs21@mails.tsinghua.edu.cn

**Keywords:** limestone calcined clay cement, excavated spoil, chloride, carbonation, durability

## Abstract

To validate the long-term performance of self-developed limestone calcined clay cement (LC3), this study evaluated the durability performance of LC3 produced using calcined excavated spoil. Results showed that LC3 exhibited a faster chloride adsorption rate than OPC, achieving peak binding capacity within 14 days, although its total chloride-binding capacity was slightly lower. The chloride diffusion coefficient of LC3 was approximately one order of magnitude lower than that of OPC, enhancing chloride resistance. However, LC3 demonstrated weaker carbonation resistance due to complete decomposition of portlandite (Ca(OH)_2_) and ettringite (AFt), alongside partial degradation of calcium silicate hydrate (C-S-H) gels, resulting in pore structure coarsening. Compared to LC3 made with commercial metakaolin (K0), the self-developed LC3 using K1 and K2 clays from excavated spoil showed comparable chloride-binding capacity but slightly weaker chloride penetration resistance. Its carbonation resistance surpassed K0-based LC3. Overall, the self-developed LC3 matched commercial metakaolin-based LC3 in durability, validating the use of locally sourced clays. Producing LC3 from calcined excavated spoil addresses environmental challenges associated with spoil disposal while delivering satisfactory durability.

## 1. Introduction

Limestone calcined clay cement (LC3) is an innovative, low-carbon binder engineered to address the dual challenges of sustainability and durability in construction. Composed of Portland clinker (30–50%), calcined kaolinite clay (30–40%), and limestone (15–20%), LC3 leverages the synergistic reactivity of its components to reduce the clinker content—the primary source of CO_2_ emissions in traditional cement production [1,2]. Calcined clay, produced by heating kaolinite-rich clay at 700–800 °C, becomes a highly reactive pozzolanic material, while limestone (CaCO_3_) acts as both a filler and a reactive ingredient [3,4]. During hydration, calcined clay releases silica and alumina, which react with calcium hydroxide (Ca(OH)_2_) from the clinker and limestone to form calcium silicate hydrates (C-S-H) and carboaluminate phases (e.g., hemicarboaluminate) [5,6]. This process not only enhances mechanical strength but also refines the microstructure of the concrete, reducing porosity and improving long-term durability [7]. By cutting clinker use by up to 50%, LC3 reduces CO_2_ emissions by 30–40% compared to ordinary Portland cement (OPC), positioning it as a cornerstone material for sustainable infrastructure [8,9].

The durability performance of concrete structures has attracted significant attention due to their extended service life spanning decades or even centuries [10,11]. Among the durability parameters, resistance to chloride ion penetration and carbonation resistance are recognized as the two critical indicators for evaluating long-term structural integrity [12,13]. Chloride-induced corrosion of steel reinforcement remains a major durability challenge for concrete structures exposed to marine, coastal, or de-icing salt environments, leading to severe structural degradation and economic losses [14,15,16]. Compared to concrete made with OPC, LC3 concrete achieves superior chloride resistance through two primary mechanisms: microstructural refinement and enhanced chloride immobilization [17]. The cementitious system undergoes complex pozzolanic reactions between calcined clay and lime, forming additional calcium–silicate–hydrate (C-S-H) gel and carboaluminates, which significantly reduce pore connectivity and permeability [16,18,19]. Studies have demonstrated that the chloride diffusion coefficient in LC3 mortars can be reduced by up to 97.6% compared to OPC [20], highlighting its superior ability to hinder chloride penetration. Simultaneously, the high alumina content in LC3 promotes the formation of Friedel’s salt (3CaO·Al_2_O_3_·CaCl_2_·10H_2_O), chemically binding free chlorides and minimizing their availability to initiate steel corrosion [21]. LC3 exhibits a higher chloride-binding capacity, up to 1.38 times that of OPC [20]. Despite reductions in calcium concentration due to pozzolanic reactions and in alkali ions due to their incorporation into reaction products, the pore solution pH values of LC3 mixes remained above 13 [22,23], ensuring the initial passivity of embedded steel rebar. However, the reduced alkalinity lowered the chloride threshold for corrosion initiation [24]. This adverse effect was mitigated by microstructural densification in LC3-based systems, which restricted ion movement. As a result, the corrosion initiation period was extended, and less expansive corrosion products formed on steel rebars [25,26]. LC3 was also shown to exhibit enhanced self-healing capacity and superior chloride resistance in cracked concrete [27].

In addition to chloride resistance, the carbonation resistance of LC3 remains a critical area of investigation [28]. Carbonation occurs when atmospheric CO_2_ penetrates into concrete, reacting with hydration products such as Ca(OH)_2_ and C-S-H to form calcium carbonate, which reduces alkalinity and compromises steel reinforcement protection [29,30]. Recent studies have shown that LC3’s lower Ca(OH)_2_ content [1,31], a result of its lower clinker dosage, makes it more susceptible to carbonation compared to OPC. This vulnerability is further exacerbated by the increased porosity and reduced alkalinity observed in LC3-based systems, which can accelerate the carbonation process and compromise the material’s long-term durability [32]. In line with a larger carbonation depth as compared to OPC-based mixes, LC3 concrete showed a significant reduction in the pore solution pH, which could limit its service life under carbonation consideration [33]. Moreover, when carbonation and chloride intrusion occur simultaneously, the decomposition of AFm phases and decalcification of C-(A-)S-H under carbonation reduces the chloride-binding ability of LC3-based composites [34]. Carbonated LC3-based composites are thus postulated to be adversely prone to corrosion in chloride-contaminated environments, such as under dry–wet cycling conditions [35]. Wang et al. [36] observed a notable increase and redistribution of free Cl^−^ in chloride-blended environments (e.g., seawater or sea-sand concrete [37]) progressing with carbonation duration, with reinforcement steel depassivation occurring at the early carbonation stage even before CO_2_ reached the steel surface.

Our previous study [3] developed a water-washing method to process the excavated spoil, yielding clay with a high kaolin content of 72.4–83.1%. The calcined clay derived from this process was incorporated into LC3, demonstrating significant pozzolanic reactivity, with compressive strength comparable to OPC paste. However, the long-term durability of LC3 produced via this method, particularly its resistance to chloride penetration and carbonation, remains insufficiently explored, hindering comprehensive evaluation of its lifecycle performance under field conditions. Therefore, experimental research was conducted in this study to assess chloride penetration resistance through chloride-binding capacity and chloride diffusion coefficient, as well as carbonation resistance via carbonation rate and microstructural analysis. The findings aim to establish a technical foundation for deploying LC3 concrete with robust durability. By innovatively repurposing calcined excavated spoil, this work develops a low-carbon LC3 material that demonstrates comparable chloride resistance and enhanced carbonation resistance relative to commercial metakaolin-based LC3. This approach pioneers the sustainable reuse of construction waste, simultaneously resolving spoil disposal challenges, reducing reliance on conventional materials, and delivering eco-friendly durability performance.

## 2. Materials and Methods

### 2.1. Raw Materials

**Figure 1:**
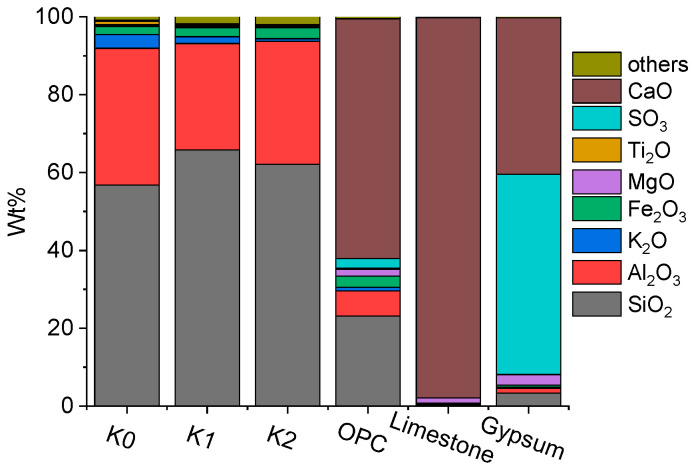
Oxide compositions of raw materials (wt %).

Two kinds of spoils, designated as K1 and K2, were excavated from the western and central regions of Shenzhen, China, respectively, and were used to prepare the LC3 paste. The spoils were washed and calcined under 800 °C for 2 h to activate their pozzolanic activity, serving as the calcined clay [3]. A commercially available calcined kaolin tailing, denoted as K0, with a high content of metakaolin, was employed for comparison to evaluate the pozzolanic activity of the aforementioned calcined spoils. The OPC employed in this study was provided by China United Cement Group Co., Ltd., Beijing, China. LC3 was produced by thorough mixing 50% cement, 33.75% calcined clay, 11.25% limestone, and 5% gypsum. The details of raw materials are shown in Figure 1, which are the same as those reported in Ref. [3].

### 2.2. Chloride-Binding Capacity

To investigate the chloride-binding capacity of LC3 paste, cubic samples with dimensions of 25 mm × 25 mm × 25 mm were prepared with a cement-to-binder ratio (w/b) of 0.4. Chloride ions were blended during the paste mixing using sodium chloride as the chloride source. The blending ratios of chloride ions were set at 0%, 0.1%, 0.3%, and 1%, respectively, relative to the binder mass. OPC samples, composed of only OPC, were used as the reference. The details of the mixture proportions are provided in Table 1. LC3 was prepared with 50% cement, 30% calcined clay, 15% limestone, and 5% gypsum based on our previous study [3].

After curing at 20 °C and 95% RH for 3, 14, 28, and 56 days, the paste samples were taken out, ground to powder, and sieved through a 60-mesh sieve. Chloride titration was then carried out to test the chloride-binding capacity of the paste samples (64 samples in total).

Acid-soluble and water-soluble chloride contents were tested according to American standards ASTM C 1152/C 1152M-2020 [38] and ASTM C1218/C1218M-20 [39], respectively. To quantify the chloride-binding capacity, the water-soluble chloride was roughly taken as the free chlorides. The bound chlorides chemically or physically associated with hydration products were derived from the difference between acid-soluble and water-soluble chloride measurements. This bound chloride fraction was subsequently normalized to total chloride content using Equation (1) to calculate the binding ratio.

(1) $$R=\frac{C_{t}-C_{f}}{C_{t}}\times 100\%$$

where R (%) represents the binding ratio, $C_{t}$ and $C_{f}$ are the contents of acid-soluble and water-soluble chlorides, respectively, expressed as mass percentage of the dried sample (wt %).

### 2.3. Rapid Chloride Migration (RCM)

To evaluate the chloride penetration resistance of LC3, cylindrical mortars with a diameter of 100 mm and a height of 100 mm were prepared for RCM tests [40]. The mortar mix for LC3-K0-0, LC3-K1-0, LC3-K2-0, and OPC-0, as specified in Table 1, had the same w/b of 0.4 but a binder-to-sand ratio of 1:3. All mortar specimens were cured for 28 d, achieving 28-day compressive strengths of 36.9 MPa, 43.8 MPa, 40.6 MPa, and 48.3 MPa for LC3-K0-0, LC3-K1-0, LC3-K2-0, and OPC-0, respectively. The central section of each sample, measuring 100 mm in diameter and 50 mm in height, was cut for the RCM tests. Three parallel measurements were conducted to ensure data reliability. The power-on time was adjusted between 24 h and 96 h based on the initial current when applying a 30 V voltage. The mortar sample was then split and sprayed with AgNO_3_ solution to test the chloride migration depth (12 samples in total).

### 2.4. Accelerated Carbonation

Mortar samples, prepared with the same mixture as used in RCM tests and with dimensions of 40 mm × 40 mm × 160 mm, were used for the carbonation experiment. The faces of the mortar samples, except for two side faces (40 mm × 160 mm), were coated with the paraffin to isolate exposure to carbonation. Accelerated carbonation was performed according to the Chinese standard GT/B 50082-2009 [40], with a CO_2_ concentration of 20 ± 0.5%, relatively humidity of 70 ± 5%, and temperature of 20 °C. After carbonation for 1, 3, 7, 14, and 28 days, the carbonated mortars were taken out and sprayed with the phenolphthalein solution to indicate the carbonation depth. The carbonation depth was determined by averaging the values from both exposed side faces of three parallel samples. A total of 60 samples were used for recording the progress of carbonation depth.

**Figure 2:**
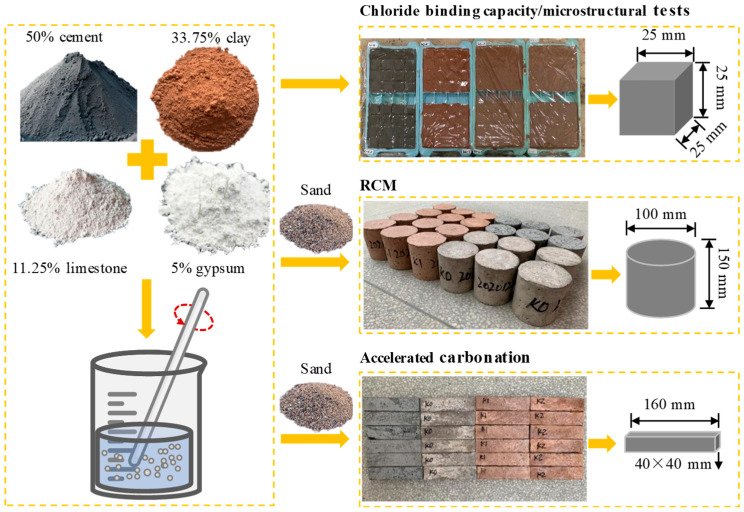
Fabricated samples used in this study.

In addition, to eliminate the effect of quartz, cement pastes with dimensions of 25 mm × 25 mm × 25 mm and the same w/b ratios (0.4) as the mortar were prepared for microstructural analysis. These cement pastes were carbonated until they reached a constant mass, with the cutting surface showing a colorless reaction upon spraying with phenolphthalein solution. The fabricated samples used in this study are illustrated in Figure 2.

### 2.5. Microstructural Analyses

For X-ray diffraction (XRD) and thermogravimetric analysis (TGA), both carbonated and uncarbonated cement pastes were ground into a fine powder using a mortar and pestle, then sieved through a 180-mesh sieve. XRD measurements were conducted using a Bruker D8 Advance diffractometer with a scanning range of 5° to 70° and a scanning step size of 0.02° per 0.1 s. TGA was performed using a simultaneous thermal analyzer (SQT Q600, TA instruments, New Castle, DE, USA) from 30 °C to 900 °C, with a heating rate of 10 °C/min under N_2_ atmosphere. The contents of portlandite ($m_{Ca{\left(OH\right)}_{2}}\%$) and calcite ($m_{CaCO_{3}}\%$) in the samples were calculated by Equations (2) and (3), respectively.

(2) $$m_{Ca{\left(OH\right)}_{2}}\%={∆m}_{385-440\;{}^{\circ}C}\times \frac{74}{18}\times 100\%$$

(3) $$m_{CaCO_{3}}\%={∆m}_{440-800\;{}^{\circ}C}\times \frac{100}{44}\times 100\%$$

where ${∆m}_{385-440\;{}^{\circ}C}$ and ${∆m}_{440-800\;{}^{\circ}C}$ are the mass losses from 385 °C to 440 °C and 440 °C to 800 °C, respectively, as indicated by the TGA curve.

For mercury intrusion porosimetry (MIP), cubic cement pastes with dimensions of 10 mm × 10 mm × 10 mm were pre-dried at 50 °C. The porosity was measured using an automatic mercury porosimeter (Autopore IV 9500, Micromeritics, Norcross, GA, USA) with a pore size range of 5 nm to 10,000 nm.

Note that, since the carbonation of OPC pastes have been extensively studied, only the microstructural analysis of LC3 pastes were conducted and compared in this study.

## 3. Results

### 3.1. Chloride Penetration Resistance

**Figure 3:**
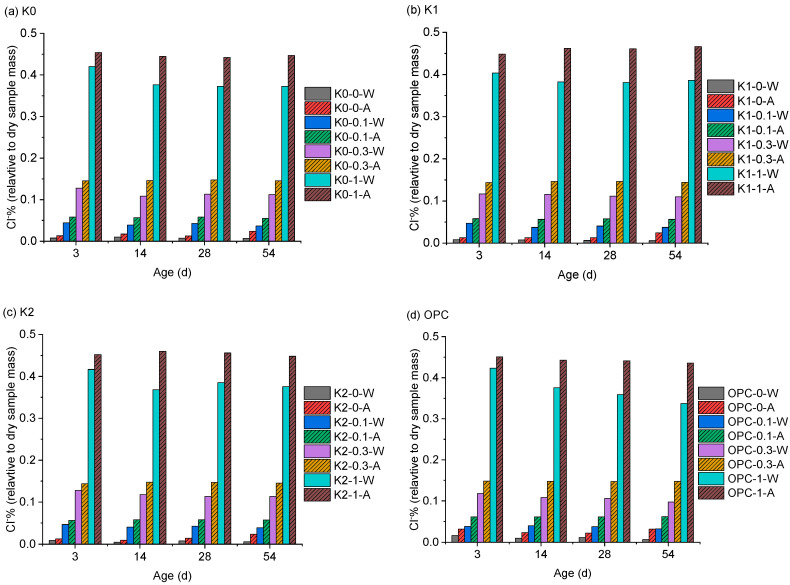
Results of chloride titration (suffix W represents water-soluble and suffix A represents acid-soluble).

**Figure 4:**
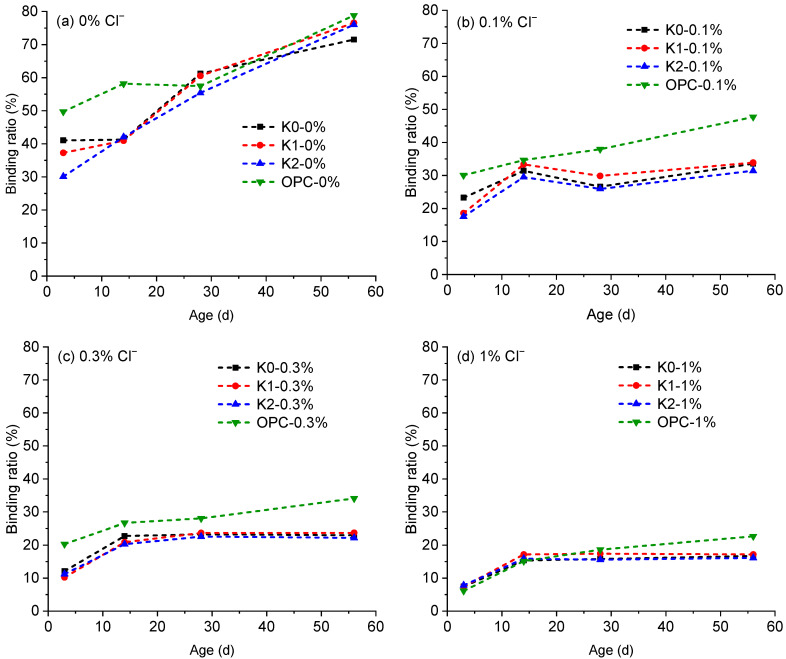
Chloride binding ratio calculated from the titration results.

The results of chloride titration are presented in Figure 3. The chloride-binding ratios, calculated from the water-soluble and acid-soluble chlorides by Equation (1), are depicted in Figure 4. Although no external chlorides were blended, trace amounts of chlorides (<0.03%) were detected under 0% Cl^−^ conditions, attributed to minor chloride content in raw materials, such as cement and clay. For all samples, the chloride-binding ratio under 0% Cl^−^ condition continuously increased with prolonged hydration age until 56 d.

Upon the addition of NaCl, the binding ratio of OPC samples continued to rise from 3 d to 56 d of hydration. In contrast, for LC3 pastes, the binding ratios only increased from 3 d to 14 d of hydration and then stabilized, reaching maximum values of 33%, 23%, and 17% when blended with 0.1%, 0.3%, and 1% of Cl^−^%, respectively, after 14 d of hydration. This indicates that LC3 paste has a higher chloride-binding capacity than OPC paste, which is advantageous in practical engineering applications, as more chlorides can be bound in a short time, thereby reducing the corrosion risk for internal steel reinforcement. Despite the use of different clays, the binding ratios of self-made LC3 pastes (K1 and K2) were comparable to those of commercially-available LC3 (K0) at the same hydration ages, demonstrating the high pozzolanic activity of the calcined clay developed in this study. However, it should be noted that beyond 14 d of hydration, the binding ratios of OPC samples were consistently higher than those of LC3 samples. Specifically, at 56 d of hydration, the chloride binding of OPC samples was approximately 14%, 12%, and 6% higher than that of LC3 paste when blended with 0.1%, 0.3%, and 1% of Cl^−^%, respectively. Therefore, while LC3 paste exhibits a higher binding rate during early hydration, its binding capacity is weaker than that of OPC paste during prolonged hydration.

**Figure 5:**
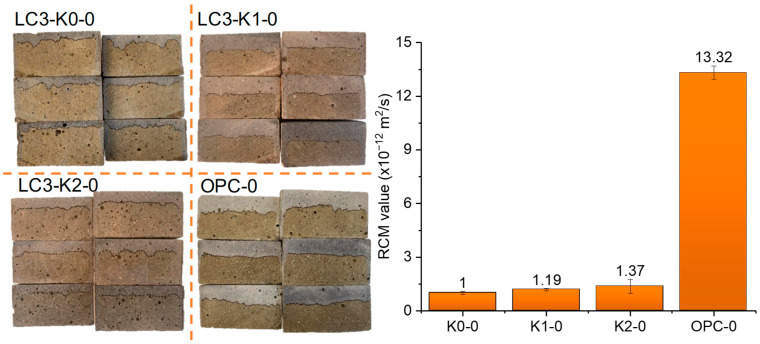
Chloride penetration profile after rapid migration (**left**) and the calculated chloride migration coefficient (**right**).

Figure 5 presents the chloride penetration profile following rapid migration, along with the calculated chloride migration coefficients. The chloride migration coefficients follow the order K0-0 < K1-0 < K2-0 < OPC-0. It is evident that the chloride transport in the three LC3 mortars is quite close (1.2 ± 0.2 × 10^−12^ m^2^/s) and about one order of magnitude lower than that in the OPC mortar (13.32 × 10^−12^ m^2^/s). The extremely low chloride migration coefficient of the LC3 mortar is expected and could be attributed to the refined pores and reduced porosity in the LC3 system [7,17].

### 3.2. Carbonation Resistance

**Figure 6:**
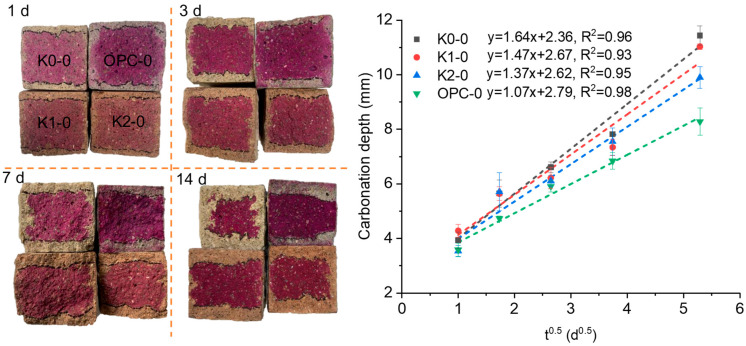
Illustration of carbonation depth (**left**) and the fitted carbonation rate (**right**).

Figure 6 presents the carbonation depth after accelerated carbonation and the fitted results. The carbonation rates were determined to be 1.64 mm/d^0.5^, 1.47 mm/d^0.5^, 1.37 mm/d^0.5^, and 1.07 mm/d^0.5^, respectively, for the K0-0, K1-0, K2-0, and OPC-0, by linearly fitting the data. It is clear that the carbonation depth of the OPC mortar was lower than that of the LC3 mortars at any given carbonation age. The inferior carbonation resistance of LC3 compared to OPC materials can be attributed to the reduced portlandite following the pozzolanic reaction [1,31]. Additionally, the carbonation rates of LC3 mortars incorporating self-made calcined clay (K1-0 and K2-0) were found to be lower than those using commercially available calcined kaolin, indicating the higher pozzolanic activity of the self-made calcine clay.

**Figure 7:**
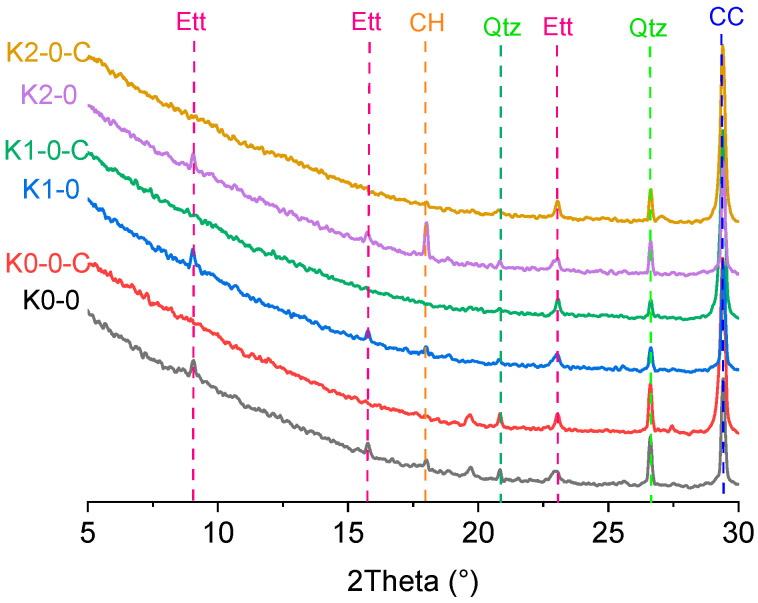
XRD patterns of LC3 pastes before and after carbonation (Note: Ett = ettringite, CH = portlandite, Qtz = quartz, CC = calcite. The suffix C represents the sample after carbonation).

To investigate the mineral transformations following carbonation, the XRD patterns of LC3 pastes before and after carbonation are presented in Figure 7. For LC3 cementitious material, portlandite was consumed via the pozzolanic reaction through its reaction with kaolinite [41]. Low portlandite peak intensities were observed in the uncarbonated K0-0 and K1-0 samples. Conversely, the K2-0 sample exhibited significantly higher portlandite intensity, which likely provides a more effective carbonation buffer. This could explain the lowest carbonation rate of K2-0 in LC3-series pastes, as depicted in Figure 6. After carbonation, the primary mineral phase transformations included the consumptions of residue portlandite and ettringite, accompanied by the formation of calcite. The XRD peaks for portlandite and ettringite disappeared entirely, indicating their complete consumption. Meanwhile, the calcite intensity increased significantly, although some peak overlap occurred due to its high intensity.

**Figure 8:**
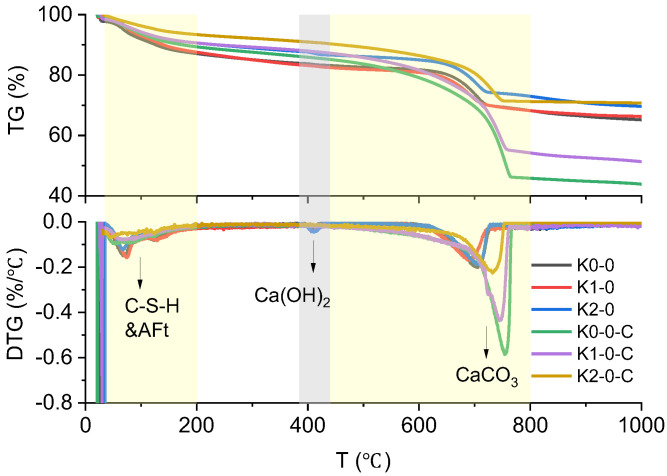
TG and it first derivative result of LC3 pastes before and after carbonation.

The TGA curves of LC3 pastes before and after carbonation are presented in Figure 8.

According to the published literature, the mass loss below 200 °C is attributed to the dehydroxylation of CSH and AFt. The mass losses between 385 °C and 440 °C, and between 440 °C and 800 °C, correspond to the decompositions of portlandite and calcite, respectively [42,43,44,45,46]. The masses of portlandite and calcite were calculated using Equations (2) and (3) and are reported in Table 2. The mass loss associated with the dehydroxylation of C-S-H and AFt phases is displayed separately, as the stoichiometric composition of CSH is variable [47].

As depicted in the results, the mass loss attributed to the decomposition of C-S-H and AFt phases decreased after carbonation, confirming their consumption during the carbonation. However, only a portion of C-S-H can be carbonated under practical conditions [48], which explains the residue dihydroxylation after carbonation. The portlandite contents in specimens K0-0, K1-0, and K2-0 were 3.0%, 3.7%, and 5.2%, respectively. These values correlate with the highest carbonation extent in K0-0 and the lowest rate in K2-0, as illustrated in Figure 6. No further mass loss from portlandite decomposition was observed after carbonation, ensuring the complete carbonation of portlandite, consistent with the XRD result in Figure 7. For calcite, approximately 30% was detected in the uncarbonated sampls introduced by initial limestone blend. Additional calcite formation in the carbonated LC3 pastes was significant: 55.4%, 42.7%, and 12.4% for K0-0-C, K1-0-C, and K2-0-C, respectively (calculated from Table 1). The low content of added calcite in K2-0-C likely stems from its weak carbonation activity.

**Figure 9:**
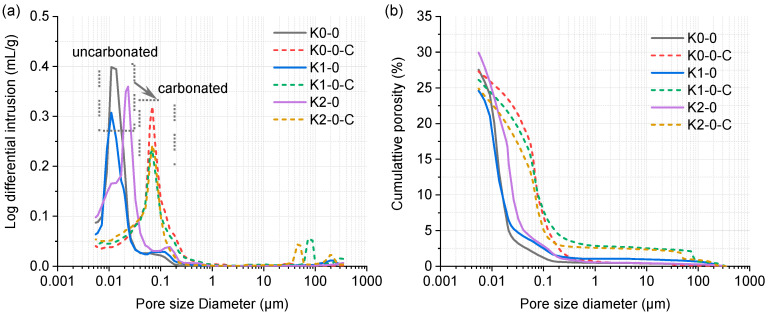
Log differential (**a**) and cumulative (**b**) pore distribution of uncarbonated and carbonated LC3 pastes.

The pore distributions of uncarbonated and carbonated LC3 pastes are shown in Figure 9. The critical pore diameter shifted rightward after carbonation. Specifically, for uncarbonated K0-0, K1-0, and K2-0, the diameters were 0.011 μm, 0.014 μm, and 0.151 μm respectively, compared to 0.069 μm for all carbonated pastes. In the LC3 system, where portlandite content is limited (Table 2), C-S-H and AFt are the dominant phases influencing porosity. Carbonation-induced volume changes—11.5% expansion for portlandite, 2.4% contraction for C-S-H, and 44.6% contraction for AFt [49]—resulted in a coarsening of the pore structure. This observation aligns with findings by Shah et al. [44]. Notably, the porosity of K2-0 decreased from 29.9% to 24.9% after carbonation, likely limiting CO_2_ ingress and explaining its low carbonation rate (Figure 6). In contrast, K0-0 and K1-0 exhibited minimal porosity changes after carbonation.

## 4. Discussion of the Durability Performance of Self-Developed LC3

The durability of concrete structures is critical due to their long service life requirements, and the LC3 system demonstrates unique advantages in this regard [31,50]. The findings of this study reveal that while the LC3 system exhibits a higher chloride-binding rate than OPC, its long-term total chloride-binding capacity is lower (see Figure 4). However, LC3’s lower chloride diffusion coefficient slows chloride ion ingress, and its ability to rapidly adsorb chloride ions after penetration—despite a slightly reduced binding ratio—enhances resistance to chloride-induced corrosion, effectively protecting internal reinforcement. This rapid chloride adsorption mechanism, previously unreported, underscores LC3’s potential for marine or chloride-rich environments

In terms of carbonation resistance, the LC3 system’s weaker carbonation performance were verified by carbonation rate and microstructural measurements, aligning with prior studies attributed to its inherently lower alkalinity [28,51]. However, this drawback is offset by its environmental and practical benefits [52]. Notably, the LC3 in this study utilized K1 and K2 clays derived from excavated spoil, which are abundant, cost-effective, and environmentally sustainable alternatives to conventional materials. This approach not only reduces reliance on high-purity commercial kaolin (e.g., K0) but also addresses waste disposal issues from construction debris.

Although LC3 samples made with K1/K2 clays showed slightly inferior chloride resistance compared to those using commercial metakaolin (K0) (see Figure 5), their chloride-binding capacity was comparable (see Figure 4), and their carbonation resistance surpassed that of K0 (see Figure 6). These results confirm that LC3 made from excavated spoil matches or outperforms commercially available metakaolin in durability aspects while offering significant cost savings. This validates the feasibility of large-scale LC3 production using locally sourced residual materials, aligning with global sustainability goals and offering a pragmatic solution for low-carbon construction.

In summary, the self-developed LC3 balances chloride resistance performance with ecological and economic advantages, positioning it as a viable and scalable alternative to traditional cement, particularly in infrastructure projects demanding long-term durability and environmental stewardship.

It should be noted that this study evaluated chloride transport and carbonation diffusion under accelerated conditions rather than in real-word conditions. The relationship between the findings of this study and their practical engineering applications requires further investigation.

## 5. Conclusions

This study investigated the durability performance of limestone calcined clay cement (LC3) produced with calcined excavated spoil, in comparison to ordinary Portland cement (OPC). The key findings are summarized as follows:

(1) Compared to OPC, the LC3 system exhibited faster chloride adsorption kinetics, reaching its maximum binding capacity within 14 days, though its total chloride-binding capacity was slightly lower. The chloride diffusion coefficient of LC3 was approximately one order of magnitude lower than that of OPC.

(2) LC3 demonstrated weaker carbonation resistance than OPC. Carbonation caused the complete decomposition of portlandite (Ca(OH)_2_) and ettringite (AFt), along with partial degradation of C-S-H gels. These chemical changes led to coarsening of the pore structure in LC3 paste.

(3) Compared to LC3 produced with commercially available metakaolin (K0), the self-developed LC3 (using K1 and K2 clays from excavated spoil) showed comparable chloride-binding capacity, albeit with slightly inferior chloride penetration resistance. However, its carbonation resistance surpassed that of K0-based LC3. Overall, the durability performance of the self-developed LC3 matched commercial metakaolin-based LC3, validating the feasibility of using locally sourced clays.

(4) Utilizing calcined clay from excavated spoil for LC3 production not only delivers satisfactory durability but also addresses environmental challenges associated with spoil disposal. This approach aligns with circular economy principles, offering a cost-effective and sustainable alternative to conventional cementitious materials.

The quantification of service life for LC3-based steel-reinforced concrete is currently under investigation by the authors and will be addressed in future work.

## Figures and Tables

**Table 1:** Mixtures of paste samples.

No.	Clay	OPC (%)	Clay (%)	Limestone (%)	Gypsum (%)	SP ^1^ (%)	Cl^− 2^ (%)
K0-0	K0	50	30	15	5	0.3	0
K0-0.1	K0	50	30	15	5	0.3	0.1
K0-0.3	K0	50	30	15	5	0.3	0.3
K0-1	K0	50	30	15	5	0.3	1
K1-0	K1	50	30	15	5	0.3	0
K1-0.1	K1	50	30	15	5	0.3	0.1
K1-0.3	K1	50	30	15	5	0.3	0.3
K1-1	K1	50	30	15	5	0.3	1
K2-0	K2	50	30	15	5	0.18	0
K2-0.1	K2	50	30	15	5	0.18	0.1
K2-0.3	K2	50	30	15	5	0.18	0.3
K2-1	K2	50	30	15	5	0.18	1
OPC-0	-	100	/	/	/	/	0
OPC-0.1	-	100	/	/	/	/	0.1
OPC-0.3	-	100	/	/	/	/	0.3
OPC-1	-	100	/	/	/	/	1

^1^ SP = polycarboxylate superplasticizer, relative to binder mass. ^2^ Cl^−^ = content of added chlorides, relative to binder mass.

**Table 2:** Calculated contents of hydration and carbonation phases from TGA results.

No.	C-S-H & AFt Related Loss (%)	Ca(OH)_2_ (%)	CaCO_3_ (%)
K0-0	4.4	3.0	34.0
K0-0-C	3.9	0	89.4
K1-0	5.0	3.7	32.4
K1-0-C	3.3	0	75.1
K2-0	3.3	5.2	31.1
K2-0-C	2.7	0	43.4

## Data Availability

The original contributions presented in this study are included in the article. Further inquiries can be directed to the corresponding author.
